# Fecal microbiota and microbial community transplantation: **a** review of current research

**DOI:** 10.3389/fmicb.2026.1814047

**Published:** 2026-06-05

**Authors:** Vitaly Ryazanov, Irina Vershinina, Ksenia Inchagova, Elena Bukareva, Vladimir Kolpakov, Alexey Ruchay, Dianna Kosyan, Maxim Marinchev, Alexey Zdorov

**Affiliations:** 1 Federal Research Centre of Biological Systems and Agro-Technologies of the Russian Academy of Sciences, Orenburg, Russia; 2 Rossijskij Universitet Druzby Narodov, Moscow, Russia

**Keywords:** aquaculture microbiome, engineered microbial consortia, fecal microbiota transplantation, FMT, synthetic microbiome

## Abstract

Homeostasis across diverse ecosystems, ranging from human hosts to environmental matrices, is profoundly governed by microbial communities. Dysbiosis, the disruption of this microbial equilibrium, leads to significant adverse outcomes in medicine, agriculture, aquaculture, and environmental health. This review synthesizes current knowledge on microbial transfusions, defined as the deliberate transfer of microbial communities or their components to restore, reconstitute, or enhance functional capacities across these systems. We explore the historical context and cutting-edge applications, including Fecal Microbiota Transplantation (FMT) for *Clostridioides difficile* infection, metabolic and neurological disorders, alongside advancements in vaginal microbiota transplantation. In agriculture, we summarize the engineering of soil microbiomes for enhanced plant health, stress adaptation, and bioremediation, as well as transplantation practices in livestock and wild species. Furthermore, we discuss the role of microbiota transplantation in aquaculture for improving fish health and disease resistance, highlighting both natural and synthetic consortia. The application of microbial communities in bioremediation and ecological restoration is explored, addressing challenges such as stability, cost, and ecological impacts. Ultimately, this review integrates these diverse applications within a “One Health” framework, emphasizing the systemic links among human, animal, and environmental microbiomes. We underscore the potential of microbiota transplantation as a sustainable strategy for restoring ecological balance while identifying critical research gaps and future directions regarding standardized methodologies and the long-term functionality of transplanted microbiomes.

## 1 Introduction

Homeostasis, the fundamental dynamic equilibrium within biological systems, is critically mediated by microbial communities across all scales, from individual organisms to entire ecosystems (Manzanera, 2025). The intricate interplay between macro- and microorganisms, as exemplified by the gut microbiota of humans and animals (Wang X. et al., 2025), the plant rhizosphere (Hnini et al., 2025), and aquatic microbial communities (Thukral et al., 2023), is essential for maintaining stability and function.

**Figure 1:**
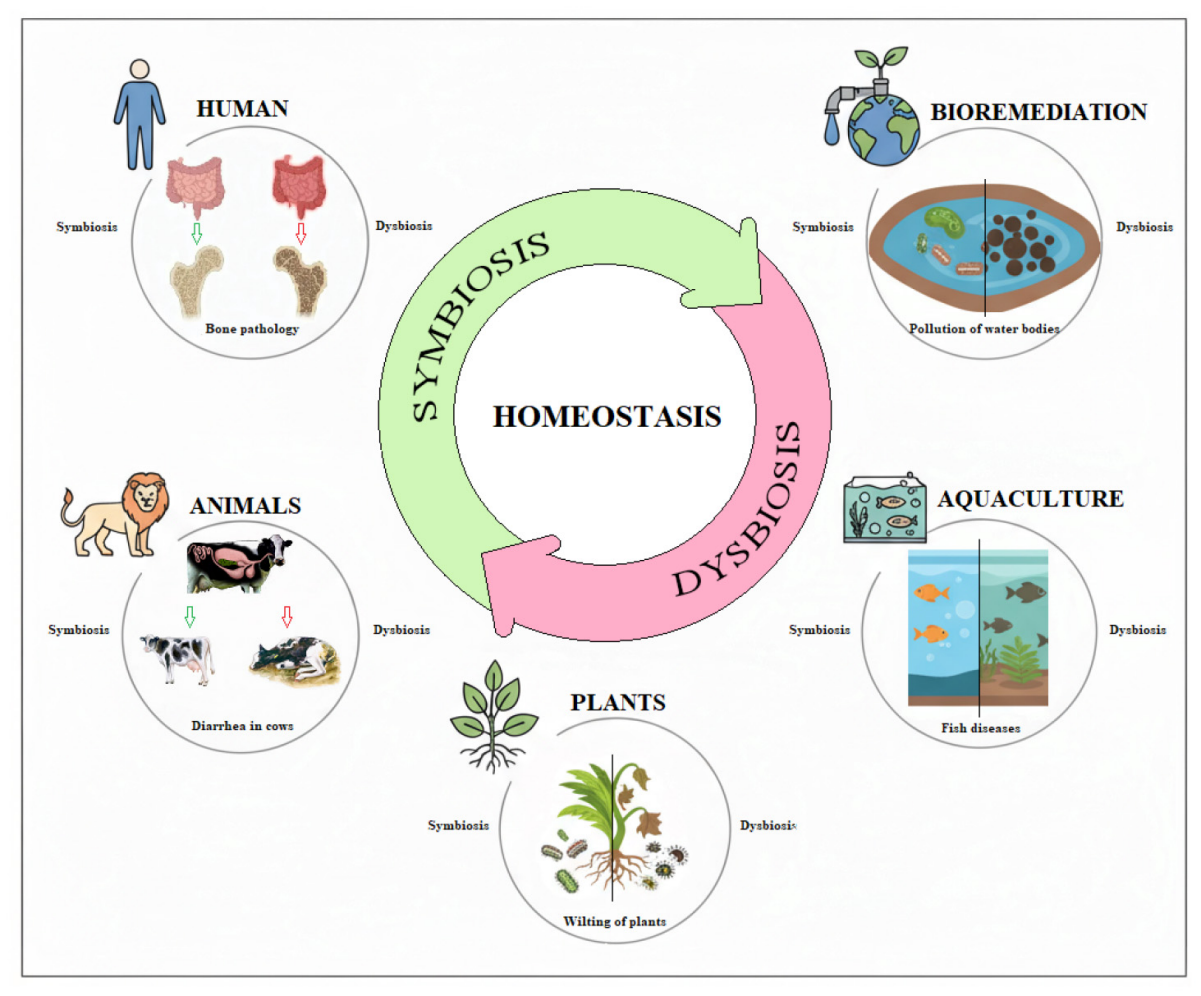
The intricate interplay between macro- and microorganisms, encompassing both symbiotic and dysbiotic interaction states.

However, detrimental factors such as antibiotic exposure, environmental stressors, and anthropogenic pollution can disrupt this delicate balance, leading to dysbiosis (Diambra, 2025). The consequences of dysbiosis are far-reaching, manifesting as disease in humans and animals, crop loss, and ecological degradation in soils and aquatic environments (Figure 1).

The restoration of microbial balance in dysbiotic ecosystems has emerged as a highly relevant and promising research area. While targeted microbial interventions have gained traction within specific disciplines, such as fecal microbiota transplantation in medicine, a comprehensive, cross-domain synthesis is critically needed to identify overarching principles, transferable technologies, and shared challenges that transcend disciplinary boundaries (Eren and Banfield, 2024; Kamble, 2024; Soth et al., 2025). Existing reviews have focused predominantly on single applications (e.g., FMT in medicine) or specific environmental contexts (restoration of disturbed soils; increasing plant yields by introducing PGPR (plant growth promoting rhizobacteria); wastewater recycling, and similar interventions). This has resulted in a significant gap in the literature regarding the pervasive nature and conceptual commonalities of microbial interventions across diverse ecosystems (Soth et al., 2025). This review explicitly addresses this gap by offering a holistic perspective on “microbial transfusions” as a unified conceptual framework, demonstrating how fundamental principles of microbial community restoration are universally applicable (Song, 2023).

We define “microbial transfusions” as the deliberate and targeted transfer of microbial communities—or their specific components—to reconstitute, restore, or enhance the functional capacity of a recipient microbiome. This definition emphasizes the therapeutic or ameliorative intent, focusing on the functional output and systemic impact of the transplanted microorganisms, rather than solely their source (e.g., fecal matter, soil, synthetic consortia) (Albright et al., 2021; Custer et al., 2023). This conceptual shift enables a unified discussion of diverse applications, fostering cross-disciplinary learning and innovation, and influencing research directions toward more harmonized and effective solutions.

The described approach to “microbiota transplantation” directly aligns with the principles of “One Health.” By focusing on the functional outcome of microbial transplantation, we recognize their role in the complex network of interactions between microbiomes in different environments. This allows us to move from targeted interventions to holistic strategies for preserving and improving microbiomes at all levels—from the organism to the ecosystem.

This review presents examples of microbial transfusions in human medicine, agriculture, aquaculture, and bioremediation, discusses their application in restoring environmental balance, examines associated risks, and explores the concept within the overarching “One Health” framework.

## 2 Microbial transplantation in medicine

The influence of the gut microbiota on the human body and overall wellbeing is a well-established fact (Kowal, 2025). A shift in the normal balance of “beneficial bacteria” toward an increase in opportunistic pathogens within the gut microbiota leads to adverse consequences, including diarrhea, constipation, irritable bowel syndrome, and other gastrointestinal diseases (Charitos et al., 2025; Shen, 2025), thereby reducing an individual's quality of life. Furthermore, recent studies have indicated that an imbalance in the gut microbiota may contribute to extra-intestinal diseases, including autism, cancer, and metabolic syndrome, among others. This presents a significant medical challenge, necessitating in-depth study and the development of therapeutic methods for maintaining a healthy balance of the gut microbiota (Biazzo and Deidda, 2022).

Fecal microbiota transplantation (FMT) from a healthy donor to a recipient with gut microbiota dysbiosis is an approach to treating intestinal dysbiotic conditions. The application of this method dates to ancient China (300–400 AD) and is currently experiencing a surge in popularity in research for treating various diseases associated with its imbalance. In certain countries, it is an effective therapy for *Clostridioides difficile* infection (CDI), caused by the opportunistic bacterium *C. difficile* (Wang et al., 2022) (Table 1).

**Table 1:** Clinical applications and outcomes of FMT and VMT.

Application area	Intervention type	Key research findings	Source
Recurrent CDI	Donor FMT via duodenal infusion	Therapeutic efficacy reached 94% after 1–2 infusions, significantly outperforming standard vancomycin therapy (500 mg orally, four times daily for 4 days). A concomitant increase in the *Bacteroidetes* and *Clostridia* and a decrease in *Pseudomonadota* were observed.	(Kelly et al., 2016; Van Nood et al., 2013)
Post-antibiotic gut microbiome restoration	Autologous FMT (auto-FMT)	The intervention demonstrated a more rapid and comprehensive reconstitution of the intestinal microbiome compared to commercial probiotic formulations.	(Suez et al., 2018)
Ulcerative colitis	Multi-donor FMT	Steroid-free clinical and endoscopic remission was achieved in 27% of patients in the FMT group vs. 8% in the placebo group. Microbial analysis linked higher diversity and specific taxa (e.g., genus *Fusobacterium*) to clinical outcomes.	(Paramsothy et al., 2017)
Metabolic syndrome	Lean-donor FMT	Improvement in insulin sensitivity was recorded at 6 weeks, alongside shifts in microbiota composition. However, these metabolic changes were not sustained at the 18-week follow-up.	(Kootte et al., 2017)
Obesity	Lean-donor FMT	In murine models, direct or horizontal transfer (co-housing) of “lean” microbiota prevented weight gain and the development of metabolic phenotypes in recipients harboring “obese” microbiota.	(Ridaura et al., 2013)
Hypertension management	FMT	Established a correlation between gut microbiota composition and blood pressure dynamics; specifically, a reduction in *Eggerthella lenta* and an increase in *Prevotella copri* levels were linked to blood pressure regulation.	(Fan et al., 2025; Lin et al., 2024; Zhong et al., 2021)
Microbiome recovery in oncology (allo-SCT)	Autologous FMT (auto-FMT)	Successfully restored gut microbiome composition and enhanced microbial diversity in patients following antibiotic therapy associated with allogeneic stem cell transplantation.	(Taur et al., 2018)
Parkinson's disease	FMT	Confirmed procedural safety and tolerability, with observed improvements in non-motor symptoms, including sleep quality and chronic constipation.	(DuPont et al., 2023; De Sciscio et al., 2025)
Recurrent bacterial vaginosis	Vaginal microbiota transplantation	Showed clinical improvement and successful restoration of a *Lactobacillus*-dominant vaginal microbiome in 4 out of 5 patients.	(Lev Sagie et al., 2019)

Analysis of the clinical trials presented in Table 1 highlights several key insights. First, there is a notable disparity in the efficacy of Fecal Microbiota Transplantation. Specifically, for treating recurrent *Clostridioides difficile* infection (CDI), efficacy rates ranged from 81% to 94%, significantly exceeding the results of standard vancomycin therapy as described in Van Nood et al. (2013) and Kelly et al. (2016). Conversely, when FMT was utilized for ulcerative colitis or metabolic syndrome, the effects were less pronounced, showing a 27% remission rate in ulcerative colitis (Paramsothy et al., 2017) and variable improvements in insulin sensitivity in metabolic syndrome (Kootte et al., 2017). This suggests a more complex, multifactorial etiology for these conditions.

Second, multiple studies, including those by Van Nood et al. (2013), Paramsothy et al. (2017), and Fan et al. (2025), have demonstrated that shifts in intestinal microbial diversity play a crucial role in improving clinical outcomes. These shifts often involve a reduction in the *Pseudomonadota* phylum and an increase in *Bacteroidetes* and *Clostridia*. These taxonomic changes are likely driven by the ability of *Bacteroidetes* and *Clostridia* to synthesize short-chain fatty acids such as butyrate and propionate, which exert anti-inflammatory effects (Porter and Larsbrink, 2022; Singh B. K. et al., 2023). These metabolites fulfill critical protective roles: butyrate serves as the primary energy source for colonocytes, strengthens epithelial tight junctions to restore barrier function, and suppresses inflammation by inhibiting NF-κB and promoting regulatory T-cell differentiation (Singh B. K. et al., 2023). Propionate, in turn, modulates systemic metabolic processes and suppresses inflammation via G-protein-coupled receptors (Porter and Larsbrink, 2022). Consequently, the reduction of the *Pseudomonadota* phylum, which contains many pathogenic and opportunistic taxa, is beneficial for both the gut and the overall organism (Young et al., 2026).

The high efficacy of FMT in recurrent CDI can be attributed to several synergistic mechanisms: FMT restores normal bile acid metabolism; donor bacteria, particularly those from the *Bacteroides* genus, express bile salt hydrolase enzymes that convert primary bile acids (which stimulate *C. difficile* spore germination) into secondary bile acids that do not (Lucky, 2025). Furthermore, the restoration of SCFA production, especially butyrate, not only reinforces the epithelial barrier but also directly disrupts *C. difficile* metabolic activity, inducing bacterial cell autolysis (Pilla et al., 2025). Finally, FMT modulates host innate immunity by inducing the expression of IL-22 and GM-CSF, promoting neutrophil activation and creating an environment hostile to *C. difficile* colonization (Khoruts et al., 2021).

Third, the findings of Suez et al. (2018) have shifted the paradigm of microbiome recovery, showing that commercial probiotics are less effective than autologous FMT (using the patient's own material collected before treatment) following antibiotic therapy. This challenges the universal use of probiotics and supports a personalized approach to microbiota restoration.

Fourth, the use of FMT to reconstruct the gut microbiome in oncology patients undergoing antibiotic therapy for allogeneic stem cell transplantation is critical (Taur et al., 2018), as these patients represent a high-risk group. Finally, preliminary research on FMT for treating hypertension and Parkinson's disease reveals, a clear link between the gut microbiota and these conditions, broadening our understanding of its systemic impact (Fan et al., 2025; De Sciscio et al., 2025).

In this context, clinical data on the use of Vaginal Microbiota Transplantation for recurrent bacterial vaginosis remain limited (Lev Sagie et al., 2019). However, its efficacy is increasingly discussed in literature reviews by Luo et al. (2024), Meng et al. (2024), and Tuniyazi and Zhang (2023). Furthermore, the existence of FDA-approved donor screening protocols and detailed methodologies for the collection and analysis of donor material establishes a solid foundation for its implementation in medical practice (Yockey et al., 2022).

Despite its positive aspects, FMT has several significant limitations, most notably the challenge of selecting healthy donors. A review of clinical data confirmed that the efficacy of FMT in chronic diseases (e.g., inflammatory bowel disease, type 2 diabetes) varies considerably depending on the donor. Some “super-donors” have clinical remission rates twice as high as those of other donors. This is attributed to the characteristics of their microbiota, including high diversity and the presence of “keystone species” such as butyrate-producing strains, which are critically important for immune and metabolic regulation (Wilson et al., 2019). The authors note that the “super-donor” phenomenon requires further research, including the standardization of selection criteria and long-term monitoring. For instance, the influence of viruses and fungi on FMT success remains unclear (Morales et al., 2024). Successful engraftment of donor strains after FMT primarily depends on the strain abundance in the donor and phylogenetic similarity to the recipient's microbiota. Smillie et al. (2018) demonstrated that strains of a single species colonize the recipient's gut according to an “all-or-nothing” principle.

Non-etheless, clinical data on FMT continue to accumulate, suggesting a promising trajectory toward its widespread implementation for the correction of human dysbiosis and the management of associated comorbidities in the foreseeable future. This perspective is reinforced by current comprehensive reviews addressing standardized procedural protocols. These include stringent donor selection criteria (e.g., rigorous screening for infectious agents and other pathologies), optimized material processing—notably the finding that freeze-thaw cycles do not compromise therapeutic efficacy—and diverse administration modalities, such as colonoscopy, nasogastric tubes, or oral capsules (Ooijevaar et al., 2019). Furthermore, critical discourse remains focused on safety profiles and potential risks (Cammarota et al., 2017; Bieganska et al., 2025), alongside the ethical and regulatory challenges associated with FMT commercialization and longitudinal safety (Allegretti et al., 2019; Ma et al., 2017; Daloiso et al., 2015). Additional considerations include the accessibility of the methodology (Cymbal et al., 2025) and the sustained impact of FMT on the recipient's gut microbiome architecture (Li et al., 2016). While these foundational studies primarily delineate the applications of fecal microbiota, we posit that the highlighted clinical, ethical, and technical frameworks are equally applicable to vaginal microbiota transplantation within the field of gynecology.

Microbial transfusions remain in early development, requiring long-term safety and efficacy studies. Synthetic microbial communities present a new direction, with synthetic biology advancing their design (Kumar et al., 2022; Nazir et al., 2024; Dou and Bennett, 2018). Yet clinical application depends on establishing causal host-microbiota links—complicated by interaction complexity. Further research using gnotobiotic models is therefore essential (Bolsega et al., 2021).

## 3 Microbial transplantation in agriculture

### 3.1 . Plants

Metagenome transplantation and the engineering of synthetic microbiomes have emerged as increasingly pivotal research frontiers, extending beyond human and veterinary medicine to encompass plant and edaphic ecosystems. This paradigm shift is intrinsically linked to the “One Health” framework, which recognizes the profound interdependence between the health of humans, animals, plants, and the surrounding environment, a topic which will be discussed in subsequent sections.

Substantial evidence in the current literature suggests that soil microbial communities can be strategically modified to manipulate plant traits and soil processes, thereby unlocking novel avenues for sustainable agricultural intensification. For instance, Panke-Buisse et al. (2015) investigated the influence of soil microbiomes on plant functional traits, specifically focusing on flowering phenology and productivity. Utilizing *Arabidopsis thaliana* and selected microbiomes, the authors identified specific taxa that either accelerate (e.g., *Xanthomonadaceae, Pseudomonadaceae, Moraxellaceae, Cellulomonadaceae*, and *Saprospiraceae*) or delay (e.g., *Iamiaceae, Alcaligenaceae, Corynebacteriaceae*, and *Verrucomicrobiaceae*) flowering. Their findings demonstrated that these microbiomes consistently modulated plant phenotypes across various *A. thaliana* genotypes and the related species *Brassica rapa*, with the notable exception of the Ler genotype. Furthermore, microbiomes associated with delayed flowering were linked to increased inflorescence biomass and enhanced activity of enzymes involved in soil nitrogen mineralization. Host-mediated microbiome selection represents a cornerstone of microbiome engineering, offering an innovative approach to augmenting both plant and animal health. Mueller and Sachs (2015) proposed utilizing host phenotypes as indicators to select microbial communities that confer beneficial traits, such as enhanced pathogen resistance or optimized nutrient acquisition. This methodology, rooted in artificial selection, quantitative genetics, and community ecology, facilitates the development of stable, functional microbiomes for clinical and agricultural applications. Such interventions are poised to bolster plant resilience to drought and disease, while simultaneously improving infection resistance in livestock (Ray et al., 2020). However, the authors also acknowledge significant challenges, including the complexity of predicting multi-species interactions and potential adverse ecological consequences. To optimize these methods, the implementation of the “Design-Build-Test-Learn” cycle—integrating metagenomics and synthetic biology—is recommended.

The intensive study of plant microbiome transformation and synthetic community development is driven by its critical role in mediating host adaptation to biotic and abiotic stressors. Consequently, this field has become a focal point in agricultural and ecological research. It is well-established that the composition and function of rhizosphere and phyllosphere microbial communities can determine plant resistance to pathogens, such as the Cotton Leaf Curl Virus, as well as tolerance to salinity and drought (Aqueel et al., 2023; de Bashan et al., 2012; Mendes et al., 2013).

The importance of microbiome engineering is further highlighted by its capacity to mitigate the impacts of salinity and water scarcity. Genera such as *Pseudomonas* and *Bacillus* have been shown to improve plant water status by synthesizing osmoprotectants and promoting root growth. Research on *Arabidopsis* underscores that soil microorganisms can enhance drought tolerance by modulating host hormonal signaling (Wubs et al., 2016). Furthermore, microbial communities in organic farming systems—characterized by an abundance of nitrogen-fixing and phosphorus-solubilizing bacteria—exhibit superior stress resilience compared to those in conventional systems (Hartmann et al., 2015).

Pérez Castro et al. (2019) observed that under drought conditions, microbial communities undergo metabolic shifts, transitioning from the decomposition of complex organic compounds to simpler substrates, which may ultimately diminish soil carbon use efficiency. A particularly salient aspect is the interaction between drought and invasive plant species; the latter further perturb microbial composition and activity, accelerating the decomposition of soil organic matter.

In conclusion, the development of synthetic microbiomes and the implementation of microbiome transplantation in crop science provide transformative opportunities for enhancing agricultural sustainability, overcoming climatic stressors, and pioneering innovative technologies for yield optimization.

### 3.2 . Animals

#### 3.2.1 Polygastric

Since the 17^th^ century, transfaunation (rumen fluid transfer) has been used to treat digestive disorders in cattle, such as rumen hypomotility and anorexia. This method restores the beneficial anaerobic microorganisms that normalize fermentation (Niederwerder, 2018; Chaitman et al., 2016).

Data are available summarizing the results of an experiment on calves supplemented with rumen fluid from donor cows. Results showed that the abundance of eight genera was significantly higher in the experimental group (namely, *Acidiphilium, Jeotgalibaca, Polaribacter, Pseudodesulfovibrio, Bdellovibrio, Microbacterium, Eubacterium* and *Sporosarcina*, belonging to four phyla: *Firmicutes, Proteobacteria, Bacteroidetes*, and *Actinobacteria*). Notably, the phyla *Bacteroidetes, Firmicutes*, and *Proteobacteria* were identified as the most abundant microbial phyla in the rumen of adult dairy cows. It is hypothesized that supplementation with rumen fluid may accelerate the development of a mature rumen microbial community in calves. Since the inoculum samples used for the experimental group were obtained from an adult cow, metatranscriptomic analysis provided the scientific community with compelling evidence for the successful transplantation of these adult cow-derived microbial phyla (Li et al., 2019).

Another study presented data suggesting that rumen fluid transfaunation in yaks significantly increased the alpha diversity of the fecal microbiota. Recipient animals showed increased abundance of bacteria such as *Rikenellaceae_RC9_gut_group, Bacteroides*, and *Christensenellaceae_R-7_group*. This was accompanied by an increased metabolism of terpenes and polyketones, amino acids, and energy (Zhang et al., 2025).

Discussion of the mechanisms occurring during transfaunation encompasses various aspects. For example, it is suggested that nutrients in rumen fluid from donor ruminants likely promote the growth of bacteria, protozoa, methanogens, and fungi, which is necessary for the restoration of normal microbiota (Jouany and Ushida, 1999). Inoculation with mature rumen fluid is known to consistently enrich recipients with key bacterial genera, including *Eubacterium* (*Firmicutes*), *Acidiphilium* (*Proteobacteria*), and *Polaribacter* (*Bacteroidetes*). This microbial shift results in a favorable change in fermentation patterns, specifically by increasing propionate concentrations and decreasing the acetate-to-propionate ratio (Arshad et al., 2021). Thus, transfaunation induces functional reprogramming at the community level—the introduction of exogenous microbiota disrupts existing niche equilibrium, initiating competitive exclusion, metabolic bypass, and complementary symbiosis, leading to optimized fermentation outcomes (Zhao et al., 2025).

DePeters and George (2014) found that transfaunation is effective for metabolic disorders in cows. Other researchers have confirmed its role in competitively excluding pathogens (Mandal et al., 2017).

A study by Rager et al. (2004) indicated that rumen microbiota transplantation (transfaunation) positively affected the health and productivity of cows after abomasal displacement surgery. Cows that underwent transfaunation exhibited better metabolic parameters, lower blood beta-hydroxybutyrate levels, and an optimal acetate-to-propionate ratio in the rumen. This reduced the need for additional intravenous dextrose therapy.

Another study revealed that rumen microbiota transfaunation in early-weaned Japanese Black calves altered the composition of the rumen microbial community and fermentation patterns. Although there were no significant differences in body weight or feed intake, transfaunation increased the proportions of acetate and butyrate while reducing propionic acid levels, thereby altering the ratio of short-chain fatty acids. Microbiota analysis showed increases in acetate- and butyrate-producing bacteria, *Methanomassiliicoccaceae_group10*, and *Neocallimastix*, and a decrease in *Methanomicrobium* (Takizawa et al., 2023).

Studies on the impact of transfaunation on microbiota composition and feeding efficiency in beef cattle suggest that introducing rumen fluid from adult animals changes the microbial community. This change increases the proportion of acetate- and butyrate-producing bacteria, which can improve energy metabolism. While no significant increase in productivity was observed, the shift in rumen fermentation suggests the potential of transfaunation to optimize digestion in beef breeds (Zhou et al., 2018). Increased diversity of the anaerobic bacterial community after transfaunation will promote the fermentation of complex carbohydrates (Sonnenburg and Bäckhed, 2016).

A study on small ruminants found that protozoal inoculation increased volatile fatty acid production, altered microbiota composition, and reduced methane emissions. However, the addition of total fauna resulted in more complex changes, including an increase in methanogens. The study highlights the key role of protozoa in regulating rumen metabolism and their potential for controlling methane emissions in ruminants. These data are important for developing strategies to reduce the environmental footprint of animal husbandry (Belanche et al., 2015). In contrast, another study investigating the long-term effects of defaunation (the removal of protozoa from the rumen) on digestion and methane production in ewes found no effect on dry matter digestibility or methane emissions, though it increased microbial protein flow and altered the volatile fatty acid profile (Bird et al., 2008).

#### 3.2.2 Monogastric

When evaluating disease prevention in monogastric animals, one study found that transfaunation reduced the incidence of porcine circovirus-associated diseases in weaned piglets. This method is also used to stimulate immunity by transferring maternal antibodies through milk (Niederwerder et al., 2018). Another study demonstrated that fecal microbiota transplantation accelerates recovery from diarrhea in puppies with canine parvovirus infection and shortens hospitalization time (Pereira et al., 2018). However, it does not affect mortality rates (Chaitman et al., 2016). The procedure was safe and well tolerated, thereby making it a promising addition to standard therapy (Khanna et al., 2017). The microbial community has coevolved with host immunity and plays a crucial role in maintaining homeostasis (Kogut et al., 2020). The microbiota stimulates the development of immune cells, such as macrophages, dendritic cells, and IgA-producing B cells, which provide protection against pathogens, including *Salmonella* (Yoo et al., 2020). Conversely, the immune system regulates microbiota composition through mechanisms of innate and adaptive immunity, such as Toll-like receptor activation and cytokine production. Disruption of this balance can lead to dysbiosis, increasing susceptibility to infections and inflammatory diseases (Zheng et al., 2020). Rumen fluid transfer in horses is a promising yet understudied method. Despite the lack of rigorous clinical evidence, preliminary data and successful applications in other species suggest that it could be an option for treating refractory cases of dysbiosis (Mullen et al., 2018).

#### 3.2.3 Wild and endangered species

Microbiota transplantation helps wild animals adapt to new habitats and food sources (Blyton and Whisson, 2019). Studies have found that a significant portion of the microbiome in leaf-eating insects is formed directly from food. Conversely, in large herbivores such as deer, the proportion of diet-dependent microorganisms is minimal (Zhu et al., 2021). Similarly, geographical location can influence the development of the gut microbiome in insectivorous bats (Dai et al., 2024). Animals of the same species living in the wild or in captivity have different bacterial community structures that provide energy to the body (Wang X. et al., 2025). One study revealed an adaptation mechanism by which the gut microbiota of Canadian cranes responds to changes in diet and environment (Xu et al., 2023). Cross-housing different animal species, both domestic (Zhang et al., 2022) and wild (Gao et al., 2025; Gogarten et al., 2018), promotes the formation of unique microbial communities through horizontal microbiota transplantation, increasing the homogeneity of these communities. The advantages of fecal microbiota transplantation in wild or captive animals include the control of infectious intestinal diseases and the ability to exploit new food resources after transplanting microbiota capable of digesting them. However, there are significant challenges in collecting feces for research, including the liquid or semi-liquid fecal consistency and as well as the practical challenges of collecting samples from healthy and diseased animals, especially for aquatic species. Taken together, these studies highlight the potential of fecal microbiota transplantation as a non-invasive strategy for improving the health, adaptability, and wellbeing of captive or endangered animals.

For example, in a study (Linnehan et al., 2024), fecal microbiota transplantation in bottlenose dolphins (*Tursiops truncatus*) under controlled conditions showed promise as a therapeutic tool for restoring gut microbiota without the use of antibiotics. In a dolphin exhibiting clinical symptoms during the study, 62 of the 205 introduced taxa (30%) showed engraftment in the experiment. The lowest percentage similarity between samples before and after FMT was also observed, indicating the greatest changes induced by fecal microbiota transplantation therapy. Prior to fecal microbiota transplantation, the dolphin's intestine was dominated by three bacterial species: *Paeniclostridium sordellii* (50.00% relative abundance), *C. perfringens* (20.37% relative abundance), and *Photobacterium damselae* (16.92% relative abundance), all of which are potential pathogens. Immediately after the first fecal microbiota transplant, an increase in the alpha diversity of the recipient's gut community and a sharp decrease in the relative abundance of these three species were observed. This coincided with significant clinical improvements in the dolphin's dysbiosis symptoms, such as improved appetite, decreased nausea, and reduced diarrhea.

An example of fecal microbiota transplantation can be found in cases of coprophagy in the wild. It is believed, for example, that consuming the feces of their chicks helps adult precocial birds stabilize their gut microbiota. This proposed mechanism may enhance microbial metabolism and help maintain energy balance in the host (Hu et al., 2022). However, it is important to note that this idea, while of considerable scientific interest, is currently purely speculative and requires substantial empirical confirmation. Existing data remains fragmentary and does not allow for definitive conclusions; further research is needed to verify the hypotheses. Fecal transplantation, achieved through coprophagy or oral administration of specific bacteria, is also being explored as a potential method for improving animal health in veterinary medicine and wildlife conservation (Bornbusch et al., 2021).

For example, it has been shown that consuming feces can help herbivores (e.g., ostriches) maintain the diversity and functionality of their gastrointestinal microbiome. The microbiomes of chickens administered fecal supplements differed not only in community composition but also in phylogenetic structure, and taxonomic abundance. 1 week after the start of the experiment, 318 differentially abundant amplicons were detected in chickens fed the fecal supplement compared to the control. Two-thirds of all these differentially abundant taxonomic units (65.8%) were more abundant in the groups supplemented with the fecal supplement compared to the controls, consistent with their higher alpha diversity. However, taxa typically associated with young age in ostriches (e.g., *Akkermansia, Blautia*, and *Dorea*) were significantly underrepresented in chickens fed the fecal supplement. For example, *Akkermansia muciniphila* was highly prevalent in control chicks at 1 week of age, while it was completely absent from chicks of the same age in the groups receiving a fecal supplement. Conversely, bacterial genera closely associated with adults, such as *Oscillospira, Treponema, Methanobrevibacter*, BF311, and YRC22, were more abundant in chicks receiving fecal supplements than in controls throughout the experiment (Videvall et al., 2023).

Thus, coprophagy in the wild leads to accelerated microbiota maturation, increased diversity, reduced pathogen burden, and enhanced growth and survival, indicating its importance for the development of young animals and the transmission of microorganisms. On the other hand, coprophagy may lead to the transmission of pathogens.

## 4 Microbial transplantation in aquaculture

The gut microbiome of fish significantly influences growth characteristics, metabolism, feeding behavior, disease resistance, and immune response (Yukgehnaish et al., 2020). When discussing the fish gut microbiota, it is important to note that its composition depends on many factors and is divided into two types: transient microbiota, which enters the body with food and does not remain for long, and resident microbiota, which permanently inhabits the body and has a stable connection with the gut (Diwan et al., 2022). The composition and quality of the habitat, geographical distribution, and species affiliation of a particular individual greatly affect the formation and functioning of the microbiota of marine and freshwater inhabitants. The results showed that habitat and host species are the primary determinants of the fish gut microbiome structure (Kanika et al., 2025).

One approach involves using probiotic strains isolated from healthy fish, or fecal microbiota transplantation from a donor to a recipient. These studies are being conducted worldwide and are gaining increasing momentum each year. For instance, Sequeiros et al. (2015). demonstrated that the *Lactococcus lactis* TW34 strain, isolated from marine fish microbiota, can produce the bacteriocin nisin Z, which inhibits the growth of the fish pathogen *Lactococcus garvieae*. This pathogen causes hemorrhagic septicemia in marine fish of the Far East, particularly in rainbow trout, Japanese amberjack, cobia, and gray mullet. Another study identified that a bacterial isolation from the gut microbiota of a deep-sea shark, presumably *Bacillus amyloliquefaciens*, can produce an antimicrobial peptide with antibacterial activity against bacterial pathogens, including *Salmonella typhimurium, Proteus vulgaris, Clostridium perfringens*, and *Staphylococcus aureus*, among others (Bindiya et al., 2015).

In this context, fecal microbiota transplantation from a donor to a recipient seems to be a more promising method for restoring fish gut microbiota, as this method utilizes not just one “beneficial microbe” but rather an entire consortium of microorganisms with stable relationships. It is important to note that this strategy involves two approaches:

- Using naturally evolved microbiota from healthy donors to restore the dysbiotic state of recipients (El Son et al., 2025).
- Using specially developed synthetic microbial communities with known beneficial properties (Guo et al., 2023; El Son et al., 2025).

The second approach may become preferable in the future, as synthetic microbial communities could include genetically modified strains with improved functions, but this requires further careful research for practical application (Tayyab et al., 2025).

Nevertheless, both approaches enable the correction of dysbiotic conditions in aquaculture, thereby improving product quality. For example, fecal microbiota transplantation from healthy koi carp has been shown to hasten the restoration of the intestinal microbiota of recipient fish after florfenicol therapy, eliminate intestinal dysfunction, and restore normal metabolite synthesis compared to natural recovery. FMT promoted the restoration of microbiota species richness and diversity by restoring populations of beneficial bacteria that were reduced by florfenicol, such as *Romboutsia, Bacteroides, Streptococcus, Lactobacillus* and *Faecalibacterium* (Han et al., 2024). Raymo et al. (2024) demonstrated that, despite weight-normalized diets, rainbow trout larvae receiving microbiome cocktails from individuals with high filet yield showed a significant increase in somatic mass as early as 27 weeks post-hatching. Two studies by Hu et al. (2022b) examined the use of FMT in zebrafish to mitigate the effects of perfluorobutanesulfonate (PFBS), a highly toxic substance that can affect the visual system, in the context of environmental pollution. The first study demonstrated the effectiveness of transplanting feces from young individuals into older individuals, alleviating metabolic disorders caused by aging and environmental pollution (Hu et al., 2022a). The second study continued the research on using FMT to restore fish health after PFBS exposure, emphasizing the effectiveness of FMT from young individuals in mitigating the effects of PFBS toxicity (Hu et al., 2022b). Chandravanshi et al. (2025) also confirmed the potential of microbiome transfusions to improve aquaculture health and survival.

However, along with the positive effects of FMT, there are some limitations and risks. These include the risk of horizontal gene transfer, host-specific microbiome variability (which complicates the development of generalized formulas), regulatory discrepancies across different jurisdictions, and socio-economic limitations restricting access in low- and middle-income countries (Tayyab et al., 2025). Additionally, the complexity of applying this approach involves technological challenges related to introducing donor microbiota to recipients. While researchers can precisely introduce donor microbiota via a probe in experimental settings, the method must be simple and economically viable on an industrial scale. This is often achieved by treating feed with an inoculum of the transplanted microbiota or by raising fish in an environment where the inoculum has been introduced. However, the effectiveness of this method is less effective than the more precise methods (Han et al., 2024; Raymo et al., 2024; Hu et al., 2022b).

Thus, FMT is currently a promising method for correcting dysbiotic conditions in aquaculture. However, its application requires a thorough analysis of the specific problem, careful selection of the donor microbiota, and the determination of the method by which the microbiota will be introduced to the recipient.

## 5 Microbial transplantation in soils

Today, humanity faces multiple challenges associated with climate change resulting from anthropogenic impact, and the accumulation of xenobiotics in the environment. Therefore, it is highly relevant to search for methods and technologies capable of restoring the natural state and ensuring the stable functioning of ecosystems. In this section, the role of microorganisms, which participate in many biogenic processes, is crucial (Han, 2020). This paper will further present an overview of existing results on the possibilities of using microorganisms for transplantation in bioremediation and environmental management.

The scientific community is currently paying attention to microbial transplantation technology for soil restoration. This microbial approach can be a turning point in restoring degraded lands (Wubs et al., 2016).

Its application has demonstrated significant potential for restoring degraded ecosystems and mitigating the effects of climate change by increasing microbial biodiversity and improving soil health (Sun et al., 2014; Benetkova et al., 2022).

Despite existing obstacles, the microbial transplantation method for soil restoration continues to develop. For example, Silva et al. (2023), along with other researchers, demonstrated that modified *Bradyrhizobium* strains increase soybean yield by 30%, improve soil health and increase yields under certain conditions. The effectiveness of these inoculants depends largely on soil type and environmental conditions, as these factors vary significantly by region. The potential of soil microbiomes to ensure agricultural sustainability by improving plant characteristics and mitigating stressors has recently been studied using the well-established approach of rhizosphere engineering. Soil microbiome transfer has also been successfully applied to protect plants (Morella et al., 2020; Anand et al., 2021; Zhang et al., 2021). Furthermore, soil microbiomes have found applications in other sectors, such as bioremediation and microbial fuel cells (Nitisoravut and Regmi, 2017). Thus, soil microbiomes can be considered as “next-generation biological preparations” with significant potential to ensure agricultural sustainability (Mitter et al., 2019).

As shown above, the potential of individual soil microbes and their consortia has traditionally been used in various biotechnological applications. However, to circumvent the main limitation of their application, limited survival and effectiveness in real systems, it became necessary to turn to microbiomes (Ray et al., 2020). Microbiomes are expected to be more stable and viable, with tightly regulated networks of interactions contributing to their effectiveness.

This approach is currently gaining widespread adoption. Although there are several reviews dedicated to soil microbiota in the context of restoration, most of them focus on the theoretical aspects of bacterial transplantation in soils (Coban et al., 2022; Contos et al., 2021; Rawat et al., 2022).

Ecosystem restoration involves mechanisms and species-specific responses that influence soil microbiota. These factors can contribute to achieving restoration goals and restoring the natural capacity of affected ecosystems for self-recovery. Various methods can be employed to influence soil microbiota and improve restoration outcomes:

1. Traditional methods (e.g., soil inoculation);

2. Non-traditional methods (e.g., using specific microbial cultures, improving seed quality).

One way to inoculate degraded ecosystems with soil microbiota and shift the microbial community toward one more characteristic of the target ecosystem is to transfer entire soil communities in the form of intact soil patches or homogenized soil. This essentially involves collecting soil from a reference ecosystem and transferring it directly to the restoration site (Koziol et al., 2018; Wubs et al., 2016). For example, Wubs et al. (2019) demonstrated that applying soil inoculants can have a long-term impact on the ecosystem, guiding and maintaining successional changes for at least two decades. However, Gerrits et al. (2023) reported that the direction of this effect depends on the suitability or compatibility of the translocated soil with the recipient site. Incompatibility can lead to disruption of microbial communities and changes in processes occurring in the soil.

Significant knowledge gaps persist regarding the effectiveness of soil translocation, including which methods are most effective (e.g., whole soil translocation, intact sod translocation, required volumes), the extent to which the physical and chemical properties of the soil at recipient sites influence establishment; how priority effects influence microbial community restoration (i.e., establishment may depend on the order of appearance of specific taxa); and how the merging of distinctly different soil communities affects successful establishment.

To overcome this limitation, a new approach has been proposed. It avoids widespread soil application and involves using local soil microbiota in the form of extruded granules or coatings as seed carriers (Gornish et al., 2019). This method can reduce the amount of soil needed by 100-fold (Stock et al., 2020). However, questions similar to those raised earlier regarding whole soil inoculation remain unresolved and are applicable here.

Increasing the availability and effectiveness of soil transplantation methods is linked to the successful implementation of natural microbiota acquisition methods. These methods can benefit key plant species used for restoration by improving seed quality (including seed extrusion and/or hydropriming with microbial additives) (Muñoz Rojas, 2018; Dadzie et al., 2022). Bioencapsulation, in which seeds are placed in protective polymers (natural and/or synthetic) that maintain inoculant viability, can also be used to enhance microbiota survival during storage and soil application (Brown et al., 2021). These methods are considered more suitable for large-scale field applications because they provide gradual and prolonged release of targeted microbiota. For example, they can be used in restoration sites to improve seed viability and introduce beneficial, selected microorganisms, such as cyanobacteria, to promote the formation of soil biocrusts (Román J et al., 2020).

Based on the aforementioned studies, it can be confidently stated that microbial communities are becoming useful tools for the bioremediation of contaminated environments. Despite their widespread use, these communities (consortia) are referred to by different terms, indicating a lack of consensus among specialists in this field (Massot et al., 2022). In studying this issue, we found that the origin of microbes and the degree of human intervention are usually considered when classifying microbiological consortia as natural (NMC), artificial (AMC), or synthetic (SMC). In this sense, NMCs are associations consisting of microorganisms derived from a single source, while members of AMCs originate from different sources. SMCs are a class of AMCs where the microbial composition is tailored for a specific task. However, researchers are questioning the existence of “gray areas” at the boundaries between each of the proposed categories of microbial consortia (Massot et al., 2022).

These initiatives have also highlighted the need to develop effective microbiome storage methods. Only a few studies have explored the concept of soil transplantation and its potential application to fresh soil microbiomes. While the application of soil microbiome transplantation is relatively new, the practice of fecal microbiome transplantation for improving human health has existed for quite some time (Kim and Gluck, 2019). Therefore, parallels can be drawn between the strategies used for fecal microbiome storage and the general strategy of preserving the microbiome in a matrix to maintain its functionality.

Soil microbiota can be manipulated to promote the restoration of degraded ecosystems by enhancing beneficial interactions between plant species and the soil microbiota, often lost in degraded environments (Aghili et al., 2014; Albornoz et al., 2022).

Based on scientific research and an analysis of existing literature, it can be concluded that microbial transplantation plays a significant role in soil restoration. Further study of this approach is required to increase its effectiveness in restoring ecological equilibrium.

Advances in DNA-based technologies and a deeper understanding of interactions between plants, soil, and ecosystems are enhancing our ability to use soil microbiota for restoration purposes (Mohr J et al., 2022). In this context, microbial transplantation represents a promising method for restoring contaminated ecosystems through the introduction of specialized microbial communities that degrade toxins, heavy metals, and organic pollutants.

The introduction of molecular genetic methods to study soil microbiome transplantation and the creation of synthetic microbial consortia has facilitated the investigation of various microbial taxa and their potential use for restoring specific ecosystem processes or relationships. For example, plant growth-promoting rhizobacteria can improve plant growth (Solans et al., 2022) and enhance germination (Domínguez Castillo et al., 2021). Chvojka (2025) presents one of the most promising new directions in creating fully synthetic microbial consortia. The study introduces Biorestorer—a systemic platform for initiating synthetic succession and synthetic pedogenesis in highly degraded or sterile substrates where natural restoration is impossible. The Biorestorer concept combines two-temperature biochar, rock-dissolving bacteria, and acidic minerals into a modular platform that reduces pH, retains water, and stimulates microbial colonization. This effectively initiates the soil formation process at an early stage. Rather than focusing on restoring natural vegetation or nutrients, Biorestorer views soil as a biotechnological interface, assembled and calibrated to operate in extreme environmental conditions. This approach is envisioned as a scalable tool for practical soil restoration, ecology, and land management in challenging environments. By combining ecological theory with engineered soil processes, this concept opens new possibilities for the accelerated restoration of soil functions and regenerative land management.

This comprehensive review has explored the multifaceted landscape of microbial transfusions, demonstrating their profound and expanding role in restoring and maintaining homeostasis across diverse biological and environmental systems. These interventions modulate human health and combat disease in medicine, enhance plant resilience and productivity in agriculture, bolster aquatic ecosystem health in aquaculture, remediate contaminated environments, and foster ecological restoration. Thus, microbial interventions represent a paradigm shift in addressing critical global challenges.

It should be noted that there are ongoing attempts to characterize the “healthy” and “unhealthy” microbiotas of plants, animals, and humans based on the Anna Karenina ecological principle (Arnault et al., 2023). In the case of soil, this principle can be described as follows: “All healthy microbiotas are alike; every unhealthy microbiota is unhealthy in its own way.” In experiments, the Anna Karenina principle often manifests in comparative analysis of various contaminations. After prolonged negative impact, experiments reveal the effect of PICT—specific resistance of soil microbiota to that impact. In various situations of soil contamination (surfactants, PAHs, petroleum products), this effect, according to researchers, also suggests a “treatment” strategy—ecological (not genetic) “editing” of the microbiome.

Based on the works summarized in this review, microbial inoculants offer significant opportunities for soil restoration and ecological farming, contributing to increased yields and plant growth (Chaudhary et al., 2022). Various artificially created bioinoculants with single or multiple microbial strains in their composition (consortium) can be used to ensure agricultural sustainability (Kumari et al., 2020; Kaur et al., 2022).

The fundamental premise underpinning these varied applications is the targeted manipulation of microbial communities to achieve desired functional outcomes (Albright et al., 2021; Custer et al., 2023). We have outlined the historical development of such practices, from ancient transfaunation techniques to cutting-edge synthetic microbial communities and underscored the transformative potential of next-generation sequencing and “omics” technologies in understanding their intricate mechanisms. The common threads of dysbiosis-driven problems and microbiome-based solutions are evident across all domains, emphasizing the interconnectedness of life on Earth.

Microbial transplantation hold promise but face challenges: standardizing donor selection, ensuring long-term stability of transferred communities, overcoming economic barriers, and addressing ethical concerns (Duhan et al., 2025; Nazir et al., 2024; Tayyab et al., 2025; Yadegar et al., 2024). Rigorous multidisciplinary research is needed to achieve mechanistic understanding amid the complexity of microbial ecosystems (Duhan et al., 2025; Lee et al., 2020).

Framed by the “One Health” concept (Lennon et al., 2023; Song, 2023), microbial transplantation represent holistic, sustainable tools rather than isolated therapies.

Future advancements require engineering synthetic consortia, using bioinformatics/AI for microbiome design, and implementing long-term monitoring. Interdisciplinary collaboration is essential to harness microbial transplantation for global health and sustainability.

## 6 Conclusion

The microbiomes of soil, plants, and animal guts play key roles in essential life processes, including nutrient cycling, stress resistance, and immunity.

The scientific community is increasingly recognizing the importance of considering soil and plant microbiomes for human health. However, the question of whether unifying beneficial or harmful functions exist across these diverse ecosystems is more complex. Current data and results remain largely indirect and hypothetical. In this regard, researchers propose integrating coevolutionary concepts, where human, soil, plant, and gut microbiomes can influence each other's evolutionary trajectories over time. This approach provides a better understanding of the exchange and interaction between soil and human gut microbiomes (Ma et al., 2025). This coevolution of microbiomes is a clear manifestation of the principles of “One Health.” This concept is based on the recognition that human health is inextricably linked to the health of soil, plants, and animals, and that microbial play a central role in this network.

The “One Health” concept emphasizes the inextricable link between human health and wellbeing and the health of other ecosystem components, such as soil, plants, and animals. Accumulating evidence indicates that microorganisms play a crucial role in the “One Health” concept by connecting these components. The health of ecosystems largely depends on the contribution of microbial communities (Berg et al., 2020). Numerous studies now demonstrate that microbial communities associated with plants, animals, and humans function as a “second genome” (Berendsen et al., 2012), an “extended genotype” (Carthey et al., 2018), or an “eco-holobiont” (Singh et al., 2020). These communities thus determine the viability and productivity of almost all organisms on Earth. Additionally, research increasingly suggests that microbial communities of different organisms are interconnected and form a closed loop (Adair et al., 2018).

Regarding the microbiome, bacterial strains partition within and between domains, forming distinct microbial communities through mechanisms described in ecology as “strain dispersal” and “ecological filtering.” Thus, the microbiomes of humans, animals, and plants comprise the “One Health Microbiome” (Wallenborn and Vonaesch, 2022), and such research should adhere to a “One Health” approach.

Taking a “One Health” approach to comprehensively study the microbiome and how it is transmitted in the environment (including the human gut) will facilitate the transfer of common concepts between different fields and accelerate translational efforts to develop targeted microbiota-based interventions. This approach, and its predictive capabilities, will enable the rational design of microbiome interventions to benefit human, animal, and environmental health (Muhummed et al., 2025).

Consequently, this review establishes a theoretical framework for advancing our understanding of how microbiome transplantation influences ecosystem resilience across both agricultural and clinical domains. By synthesizing evidence on microbiota transplantation in various environments, this work elucidates a conceptual bridge grounded in universal symbiotic principles and the restoration of ecological homeostasis, thereby highlighting critical interdisciplinary research trajectories.
